# Binge Eating, Anxiety, Depression, and personality disorder in a Clinical sample of obese Adult in Egypt

**DOI:** 10.1192/j.eurpsy.2023.1806

**Published:** 2023-07-19

**Authors:** S. K. I. M. Aboelenien, Z. Sarhan

**Affiliations:** 1 CMHT, Essex Partnership university trust (EPUT), Rochford, United Kingdom; 2 Department of Psychiatry, Faculty of Medicine, Cairo University, Cairo, Egypt

## Abstract

**Introduction:**

Obesity is a major public health problem and some developed countries have declared it ‘the modern day epidemic’. One of the major eating disorders that leads to obesity is BED, which involves consuming large quantities of high carbohydrate food. Studying the factors that cause and contribute to BED can help tackle this major health hazard and alleviate a huge burden on the nationalized health service.

**Objectives:**

To determine the frequency of Binge Eating Disorder (BED) among obese adults, and to study its relationship to depression, anxiety, life stressors, personality and self esteem.

**Methods:**

The sample was a randomised sample of clinically obese individuals, body mass index (BMI) of 30 and above. The sample was collected from two sites; Nutrition Clinic in Student’s Hospital, Cairo University and a Private Nutrition Centre. 250 cases were recruited over one year. All patients were subjected to a clinical interview derived from Kasr El Aini sheet , and measurement of Waist- Hip Ratio. Assessment of depression and anxiety was through Beck Depression Inventory , Hamilton Depression Rating Scale and Taylor Manifest Anxiety Scale(TMAS). Other tools used were the Eysenck’s Personality Inventory, Eating Disorder Inventory -2.

**Results:**

BED among obese adults was 48%; 83 % of them had drive for thinness, 25% were bulimics, 45 % had ineffectiveness feeling. Also 83 % had body dissatisfaction, 8% were perfectionism seeking, 43 % showed interpersonal distrust and 25% presented maturity fears. Impulsivity was scored high in 25% , 66.6%had social insecurity and 77% had severe Extraversion. All were statistically significant. On the other hand there were no statistical significant difference between obese adults with BED and those without on TMAS. Half percent of participants with BED and 34.6 percent of participant without BED had moderate level of anxiety. In addition , there were no significant difference between obese participants with BED and those without BED according to BDI. However, 83.3% of obese cases with BED while 60 % for those without BED had manifest depression ranging from mild to severe depression.

**Conclusions:**

Obese adults with BED have more drive for thinness, body dissatisfaction, feeling of ineffectiveness, perfectionism seeking, interpersonal distrust, maturity fears and social insecurity than non BED. Extraversion and Neuroticism are also more among BED. There were no significance different between both group in relation to Anxiety and Depression.

**Disclosure of Interest:**

None Declared

